# P02.127. Recruitment strategies for community-based yoga research in a predominant minority population

**DOI:** 10.1186/1472-6882-12-S1-P183

**Published:** 2012-06-12

**Authors:** J Keosaian, D Dresner, C Cerrada, L Kwong, N Goodman, M Tam, M Godersky, K Sherman, J Weinberg, A Boah, R Saper

**Affiliations:** 1 Boston Medical Center, Boston, USA; 2 Group Health Research Institute, Seattle, USA; 3 Boston University School of Public Health, Boston, USA

## Purpose

The Yoga Dosing Study is the first part of a 4-year NIH/NCCAM funded comparative effectiveness randomized controlled trial of yoga vs. physical therapy vs. education for chronic low back pain (CLBP) in underserved populations. We sought to recruit a diverse population by reducing barriers such as health literacy, and increasing access and awareness of research studies through community health center partnerships.

## Methods

We created a multi-faceted recruitment strategy focusing on community health centers in diverse neighborhoods in Boston. Approaches included flyers posted in waiting rooms, brochures containing culturally relevant pictures, staff and provider presentations at health centers, and targeted letters to patients with a diagnosis of low back pain. Targeted letters were made possible through the Boston HealthNet, an affiliation of Boston Medical Center with community health centers, which allows for shared access to electronic medical records. We utilized designated physicians at each clinic to act as a point person or “site champion” for the study. We will also conducted qualitative interviews on the topic of barriers to recruitment.

## Results

In four months, there were 631 inquiries about the study. Targeted letters yielded the largest number of inquires (48%). Twenty-eight percent of patients were recruited by flyers and brochures, and 13% were recruited by a physician. We enrolled 95 participants for the study; a majority was from racial or ethnic minority groups (82%). Of those who were enrolled into the study, the greatest percentage was referred by physicians (27%).

## Conclusion

A multi-dimensional recruitment strategy based on community center buy-in and support was successful and efficient. Developing and maintaining connections with community health center physicians and staff was essential for recruitment of participants. Although posted flyers and brochures yielded the most inquiries about the study, people who were recruited by their physician were more likely to enroll in the study.

